# Crystal structure and Hirshfeld surface analysis of 6-amino-8-(2,6-di­chloro­phen­yl)-1,3,4,8-tetra­hydro-2*H*-pyrido[1,2-*a*]pyrimidine-7,9-dicarbo­nitrile

**DOI:** 10.1107/S2056989021003583

**Published:** 2021-04-09

**Authors:** Farid N. Naghiyev, Tatiana A. Tereshina, Victor N. Khrustalev, Mehmet Akkurt, Rovnag M. Rzayev, Anzurat A. Akobirshoeva, İbrahim G. Mamedov

**Affiliations:** a Department of Chemistry, Baku State University, Z. Khalilov str. 23, Az, 1148 Baku, Azerbaijan; b Peoples’ Friendship University of Russia (RUDN University), Miklukho-Maklay St. 6, Moscow, 117198, Russian Federation; c N. D. Zelinsky Institute of Organic Chemistry RAS, Leninsky Prosp. 47, Moscow, 119991, Russian Federation; d Department of Physics, Faculty of Sciences, Erciyes University, 38039 Kayseri, Turkey; e "Composite Materials" Scientific Research Center, Azerbaijan State Economic University (UNEC), H. Aliyev str. 135, Az 1063, Baku, Azerbaijan; f Acad. Sci. Republ. Tadzhikistan, Kh. Yu. Yusufbekov Pamir Biol. Inst., 1 Kholdorova St, Khorog 736002, Gbao, Tajikistan

**Keywords:** crystal structure, cyclo­addition product, 1,3,4,8-tetra­hydro-2*H*-pyrido[1,2-*a*]pyrimidine, Hirshfeld surface analysis

## Abstract

In the mol­ecular structure of the title compound, C_16_H_13_Cl_2_N_5_, the 1,4-di­hydro­pyridine ring of the 1,3,4,8-tetra­hydro-2*H*-pyrido[1,2-*a*]pyrimidine ring system adopts a screw-boat conformation, while the 1,3-diazinane ring is puckered. In the crystal, inter­molecular N—H⋯N and C—H⋯N hydrogen bonds form mol­ecular sheets parallel to the (110) and (10) planes, crossing each other. Adjacent mol­ecules are further linked by C—H⋯π inter­actions, which form zigzag chains propagating parallel to [100]. A Hirshfeld surface analysis indicates that the most significant contributions to the crystal packing are from N⋯H/H⋯N (28.4%), H⋯H (24.5%), C⋯H/H⋯C (21.4%) and Cl⋯H/H⋯Cl (16.1%) contacts.

## Abstract 2

In the crystal structure of the title compound, inter­molecular N—H⋯N and C—H⋯N hydrogen bonds between the mol­ecules lead to sheets extending parallel to the (110) and (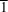
10) planes.

## Chemical context   

Chemical transformations comprising carbon–carbon and carbon–heteroatom bond-formation reactions are fundamental tools in modern synthetic organic chemistry (Yadigarov *et al.*, 2009 ▸; Abdelhamid *et al.*, 2011 ▸; Khalilov *et al.*, 2011 ▸; Yin *et al.*, 2020 ▸). They are also used for the synthesis of valuable building blocks in medicinal chemistry, coordination chemistry and material science (Mahmoudi *et al.*, 2017 ▸, 2019 ▸; Viswanathan *et al.*, 2019 ▸).

Pyrido[l,2-*a*]pyrimidines constitute a valuable class of heterocycles because many of them possess broad biological activities, such as mono­amine oxidase inhibition, anti­hypertensive, insecticide, serotonergic antagonist, analgesic, anti-inflammatory, cytoprotective, bronchodilatory, phospho­diesterase-inhibitory, anti­thrombotic, anti­allergic, anti­atherosclerotic and hypoglycaemic activities, as well as anti­tumor effects (Hermecz & Mészáros, 1988 ▸; Ukrainets *et al.*, 2018 ▸). The pyrido[1,2-*a*]pyrimidine motif is incorporated into the structure of some marketed drugs, including the anti­asthmatic agent pemirolast, the tranquilizer pirenperone, the anti­allergic agent ramastine, and the psychotropic agents risperidone and paliperidone (Awouters *et al.*, 1986 ▸; Blaton *et al.*, 1995 ▸; Yahata *et al.*, 2006 ▸; Riva *et al.*, 2011 ▸). Over recent decades, a number of synthetic protocols for the synthesis of pyrido[1,2-*a*]pyrimidines have been reported, and these approaches have focused on two-component reactions (Wu *et al.*, 2003 ▸; Pryadeina *et al.*, 2005 ▸). Multi-component reactions have developed as powerful tools for the design of complex mol­ecules, natural products and drug-like mol­ecules in a minimum number of synthetic steps (Abdelhamid *et al.*, 2014 ▸; McLaughlin *et al.*, 2014 ▸; Janssen *et al.*, 2018 ▸).
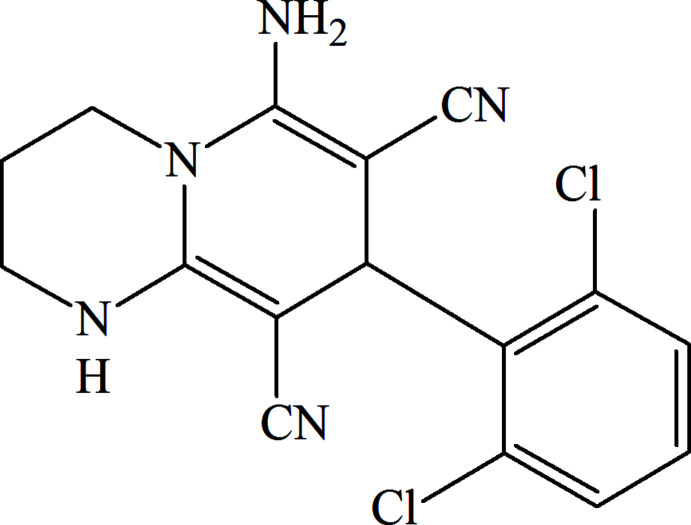


As part of our studies on the chemistry of bridgehead nitro­gen heterocycles, as well as taking into account our ongoing structural studies (Mamedov *et al.*, 2013 ▸; Naghiyev *et al.*, 2020**a* ▸,*b* ▸,*c* ▸;* Naghiyev *et al.*, 2021 ▸), we report here the crystal structure and Hirshfeld surface analysis of the title compound, C_16_H_13_Cl_2_N_5_, obtained by an efficient three-component synthetic protocol.

## Structural commentary   

**Figure 1:**
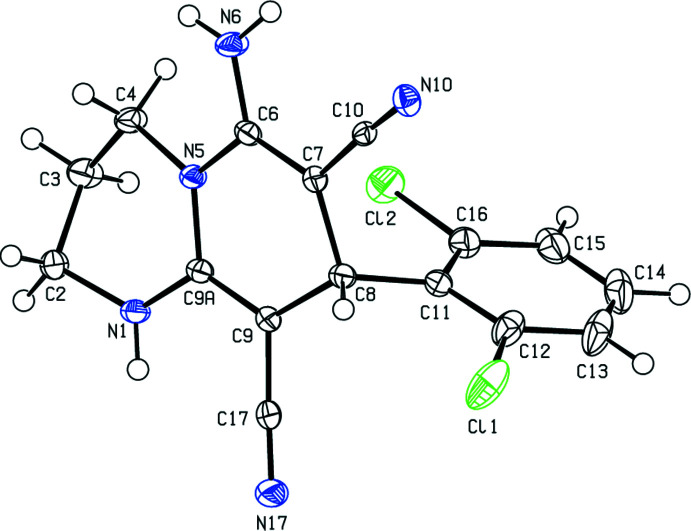
The mol­ecular structure of the title compound. Displacement ellipsoids are drawn at the 50% probability level.

In the mol­ecular structure of the title compound, (Fig. 1 ▸), the 1,4-di­hydro­pyridine ring (N5/C6–C9/C9*A*) of the 1,3,4,8-tetra­hydro-2*H*-pyrido[1,2-*a*]pyrimidine ring system (N1/C2–C4/N5/C6–C9/C9*A*) adopts a screw-boat conformation with puckering parameters (Cremer & Pople, 1975 ▸) *Q*_T_ = 0.520 (3) Å, θ = 120.8 (3)° and φ = 270.4 (3)°, while the 1,3-diazinane ring (N1/C2–C4/N5/C9*A*) is puckered [*Q*_T_ = 0.160 (3) Å, θ = 75.2 (11)° and φ = 169.4 (10)°]. The di­chloro­phenyl ring (C11–C16) makes a dihedral angle of 80.82 (12)° with the mean plane of the 1,3,4,8-tetra­hydro-2*H*-pyrido[1,2-*a*]pyrimidine ring system.

## Supra­molecular features   

**Figure 2:**
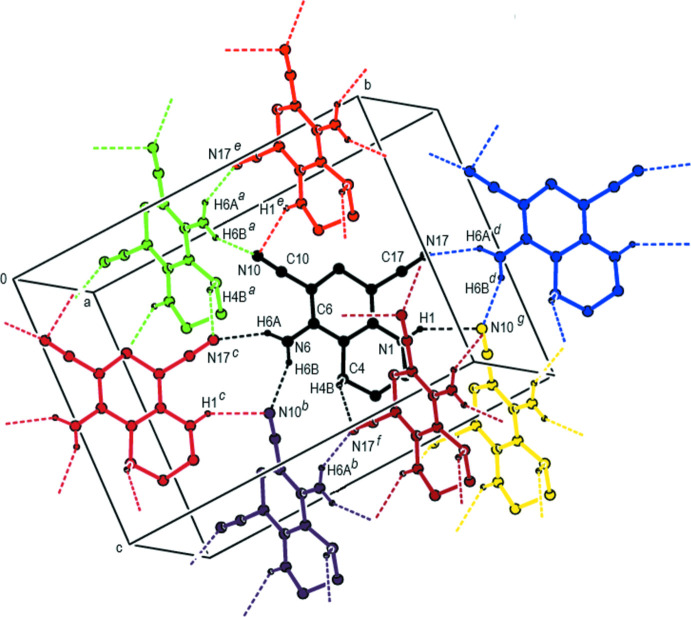
A view showing details of the inter­molecular N—H⋯N and C—H⋯N hydrogen bonds in the unit cell of the title compound. The di­chloro­phenyl group and H atoms not involved in hydrogen bonding have been omitted for clarity. [Symmetry codes: (*a*) *x*, 1 − *y*, −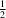 + *z*; (b) *x*, 1 − *y*, 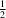 + *z*; (*c*) −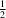 + *x*, −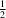 + *y*, *z*; (*d*) 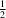 + *x*, 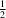 + *y*, *z*; (*e*) −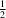 + *x*, 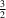 − *y*, −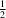 + *z*; (*f*) −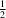 + *x*, 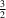 − *y*, 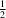 + *z*; (*g*) 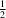 + *x*, 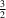 − *y*, 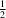 + *z*].

**Figure 5:**
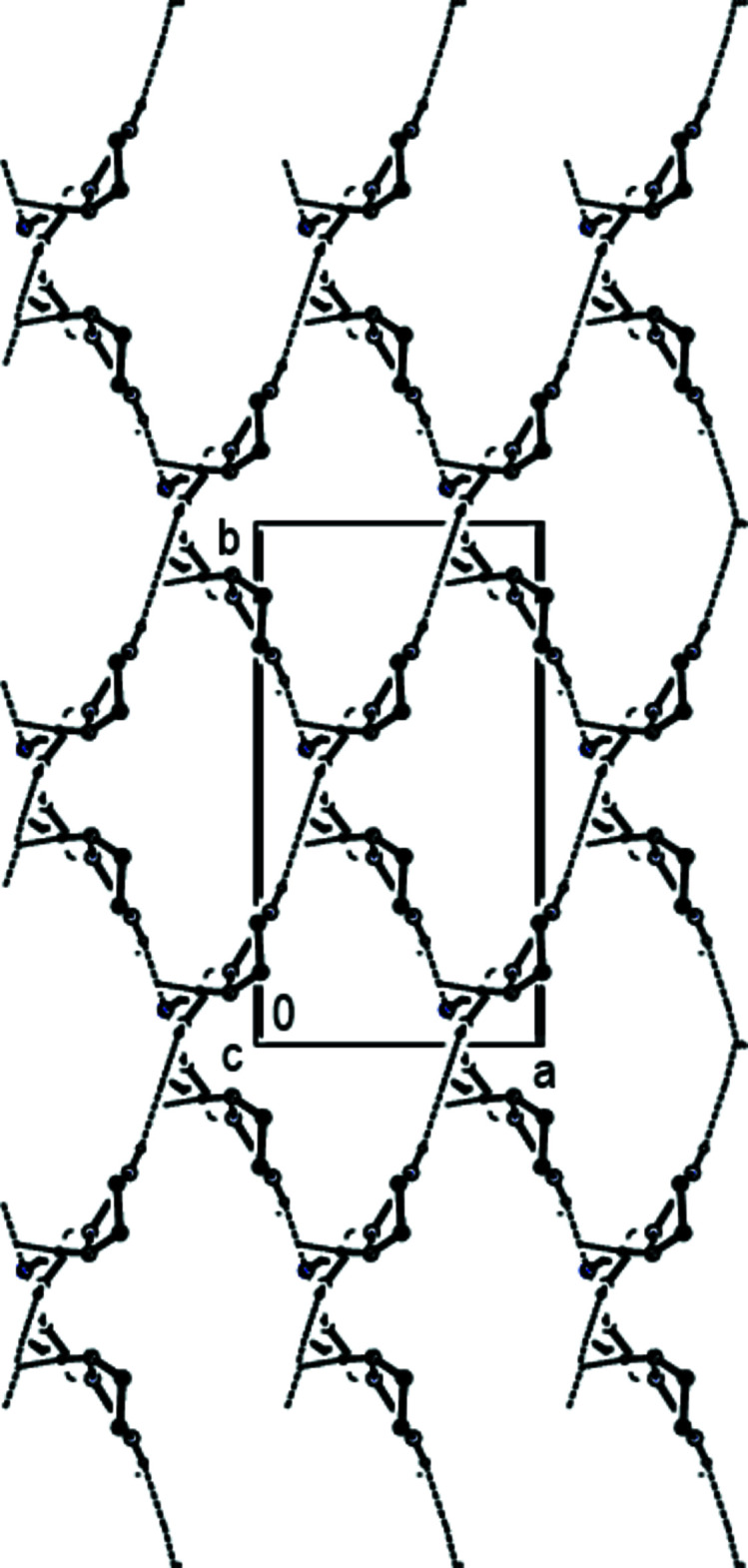
A view along [001] showing the inter­molecular N—H⋯N and C—H⋯N hydrogen bonds of the title compound. The di­chloro­phenyl group and H atoms not involved in hydrogen bonding have been omitted for clarity.

**Figure 6:**
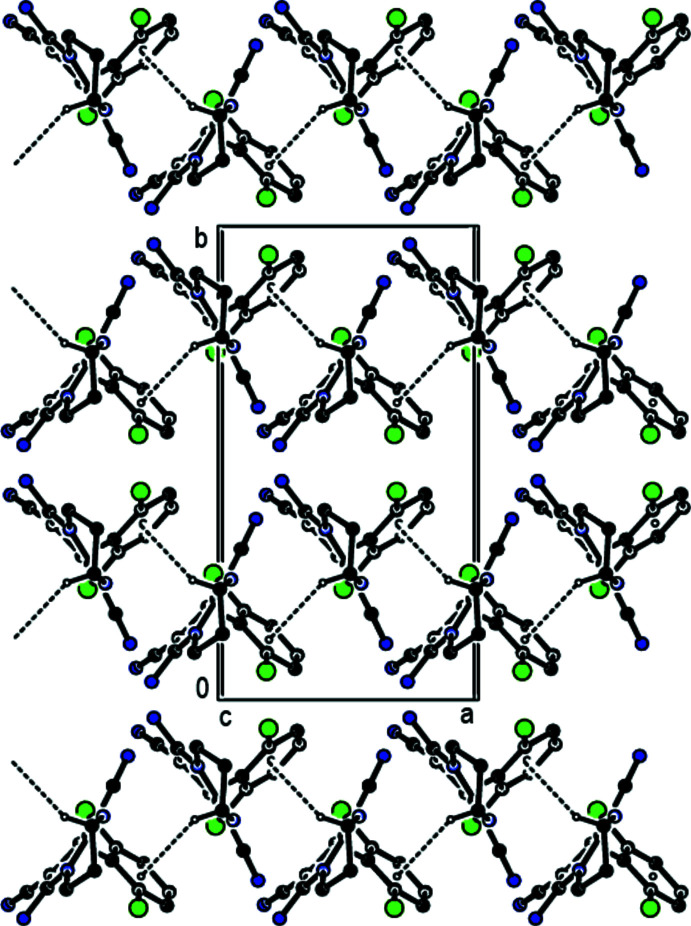
A view along [010] showing the C—H⋯π inter­actions in the title compound.

In the crystal, inter­molecular N—H⋯N hydrogen bonds between the amine functions as donor groups and the nitrile N atoms as acceptor groups and inter­molecular C—H⋯N hydrogen bonds lead to the formation of sheets extending parallel to (110) and (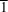
10) (Table 1 ▸; Figs. 2 ▸, 3 ▸ and 4 ▸). These hydrogen-bonded sheets cross each other (Fig. 5 ▸). C—H⋯π inter­actions (Table 1 ▸), which form zigzag chains propagating parallel to [100] (Fig. 6 ▸), are also involved in the packing.

## Hirshfeld surface analysis   

**Figure 7:**
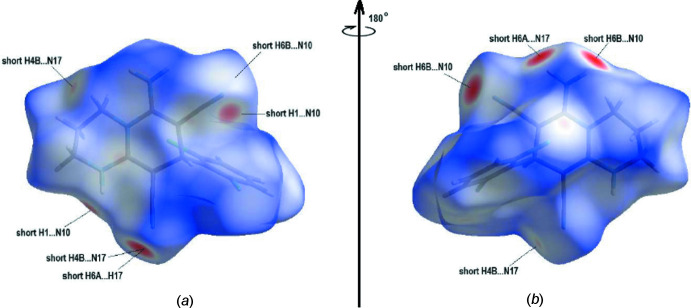
(*a*) Front and (*b*) back sides of the three-dimensional Hirshfeld surface of the title compound plotted over *d*_norm_ in the range −0.4776 to +1.4517 a.u.

In order to visualize the inter­molecular inter­actions in the crystal of the title compound, a Hirshfeld surface analysis (Spackman & Jayatilaka, 2009 ▸) was performed with *CrystalExplorer17.5* (Turner *et al.*, 2017 ▸). Fig. 7 ▸(*a*) and Fig. 7 ▸(*b*) show the front and back sides of the three-dimensional Hirshfeld surface of the title mol­ecule plotted over *d*_norm_ in the range −0.4776 to +1.4517 a.u., using a ‘high standard’ surface resolution colour-mapped over the normalized contact distance. The red, white and blue regions visible on the *d*_norm_ surfaces indicate contacts with distances shorter, longer and equal to the van der Waals separations. The red spots highlight the inter­atomic contacts, including the N—H⋯N and C—H⋯N hydrogen bonds.

**Figure 8:**
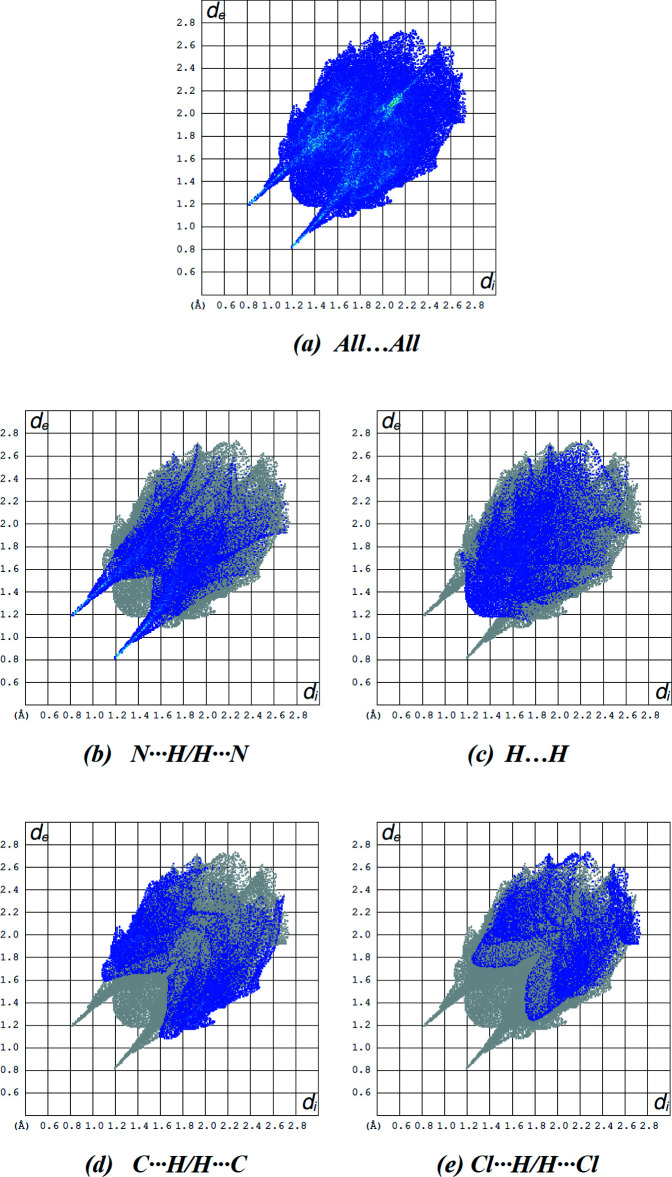
The two-dimensional fingerprint plots of the title compound, showing (*a*) all inter­actions, and delineated into (*b*) N⋯H/H⋯N, (*c*) H⋯H, (*d*) C⋯H/H⋯C and (*f*) Cl⋯H/H⋯Cl inter­actions [*d*_e_ and *d*_i_ represent the distances from a point on the Hirshfeld surface to the nearest atoms outside (external) and inside (inter­nal) the surface, respectively].

The overall two-dimensional fingerprint plot for the title compound and those delineated into N⋯H/H⋯N, H⋯H, C⋯H/H⋯C and Cl⋯H/H⋯Cl contacts are illustrated in Fig. 8 ▸. Numerical details of the various contacts are given in Table 2 ▸ and their percentage contributions to the Hirshfeld surfaces are collated in Table 3 ▸. N⋯H/H⋯N (28.4%), H⋯H (24.5%), C⋯H/H⋯C (21.4%) and Cl⋯H/H⋯Cl (16.1%) contribute significantly to the packing while Cl⋯C/C⋯Cl, Cl⋯Cl, Cl⋯N/N⋯Cl, C⋯N/N⋯C, C⋯C and N⋯N contacts have a negligible directional impact.

The large number of N⋯H/H⋯N, H⋯H, C⋯H/H⋯C and Cl⋯H/H⋯Cl inter­actions suggest that van der Waals inter­actions and hydrogen bonding play the major roles in the crystal packing (Hathwar *et al.*, 2015 ▸).

## Database survey   

Four related compounds, which have the 1,3,4,8-tetra­hydro-2*H*-pyrido[1,2-*a*]pyrimidine ring system of the title compound, were found in a search of the Cambridge Structural Database (CSD version 5.42, update of November 2020; Groom *et al.*, 2016 ▸): 9-(4-nitro­benzyl­idene)-8,9-di­hydro­pyrido[2,3-*d*]pyrrolo­[1,2-*a*]pyrimidin-5(7*H*)-one (refcode VAMBET; Khodjaniyazov & Ashurov, 2016 ▸), 11-(amino­methyl­idene)-8,9,10,11-tetra­hydro­pyrido[2′,3′:4,5]pyrimido[1,2-*a*]azepin-5(7*H*)-one (HECLUZ; Khodjaniyazov *et al.*, 2017 ▸), 7′-amino-1′*H*-spiro[cyclo­heptane-1,2′-pyrimido[4,5-*d*]pyrimidin]-4′(3′*H*)-one (LEGLIU; Chen *et al.*, 2012 ▸) and 11-(2-oxopyrrolidin-1-ylmeth­yl)-1,2,3,4,5,6,11,11a-octa­hydro­pyrido[2,1-*b*]quinazolin-6-one dihydrate (KUTPEV; Samarov *et al.*, 2010 ▸).

In the crystal of VAMBET, mol­ecules are linked *via* C—H⋯O and C—H⋯N hydrogen bonds, forming layers parallel to (101). In the mol­ecule of HECLUZ, the seven-membered penta­methyl­ene ring adopts a twist-boat conformation. In the crystal, hydrogen bonds with 16-membered ring and three chain motifs are generated by N—H⋯N and N—H⋯O contacts. The amino group is located close to the nitro­gen atoms, forming hydrogen bonds with 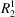
(4) and 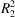
(12) graph-set motifs. This amino group also forms a hydrogen bond with the C=O oxygen atom of a mol­ecule translated parallel to [100], which links the mol­ecules into 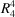
(16) rings. Hydrogen-bonded chains are formed along [100] by alternating 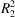
(12) and 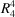
(16) rings. These chains are stabilized by inter­molecular π–π stacking inter­actions observed between the pyridine and pyrimidine rings. In LEGLIU, the mol­ecular structure is built up from two fused six-membered rings and one seven-membered ring linked through a spiro C atom. The crystal packing is stabilized by inter­molecular N—H⋯O hydrogen bonds between the two N—H groups and the ketone O atoms of the neighbouring mol­ecules. In KUTPEV, water mol­ecules are mutually O—H⋯O hydrogen bonded and form infinite chains propagating parallel to [010]. Neighbouring chains are linked by the quinazoline mol­ecules by means of O—H⋯O=C hydrogen bonds, forming a two-dimensional network.

## Synthesis and crystallization   

To a dissolved mixture of 2-(2,6-di­chloro­benzyl­idene)malono­nitrile (1.14 g; 5.1 mmol) and malono­nitrile (0.34 g; 5.2 mmol) in methanol (40 mL), 1,3-di­amino­propane (0.38 g; 5.2 mmol) was added and was stirred at room temperature for 10 min. Then 25 mL of methanol were removed from the reaction mixture that was left overnight. The precipitated crystals were separated by filtration and recrystallized from ethanol (yield 78%; m.p. 541–542 K).

^1^H NMR (300 MHz, DMSO-*d*_6_): 1.89 (*m*, 2H, CH_2_); 3.13 (*m*, 2H, CH_2_); 3.67 (*m*, 2H, CH_2_); 5.31 (*s*, 1H, CH-Ar); 6.14 (*s*, 2H, NH_2_); 6.78 (*s*, 1H, NH); 7.25 (*t*, 1H, Ar-H, ^3^ *J*_H–H_ = 7,9); 7.42 (*d*, 2H, 2Ar-H, ^3^ *J*_H–H_ = 7,8). ^13^C NMR (75 MHz, DMSO-*d*_6_): 22.30 (CH_2_), 36.32 (Ar-CH), 38.62 (CH_2_), 42.92 (CH_2_), 51.70 (=C_quar_), 55.06 (=C_quar_), 121.61 (CN), 122.04 (CN), 129.56 (3CH_arom_), 138.25 (3C_ar_), 152.11 (=C_quar_), 154.17 (=C_quar_).

## Refinement   

Crystal data, data collection and structure refinement details are summarized in Table 4 ▸. The C-bound H atoms were placed in calculated positions (C—H = 0.95–1.00 Å) and refined as riding with *U*_iso_(H) = 1.2*U*_eq_(C). All N-bound H atoms were located in a difference map [N1—H1 = 0.85 (3) Å, N6—H6*A* = 0.85 (4) Å and N6—H6*B* = 0.85 (4) Å] and they were refined with the constraint *U*_iso_(H) = 1.2*U*_eq_(N).

## Supplementary Material

Crystal structure: contains datablock(s) I. DOI: 10.1107/S2056989021003583/wm5605sup1.cif

Structure factors: contains datablock(s) I. DOI: 10.1107/S2056989021003583/wm5605Isup2.hkl

Supporting information file. DOI: 10.1107/S2056989021003583/wm5605Isup3.cml

CCDC reference: 2075706

Additional supporting information:  crystallographic information; 3D view; checkCIF report

## Figures and Tables

**Figure 3:**
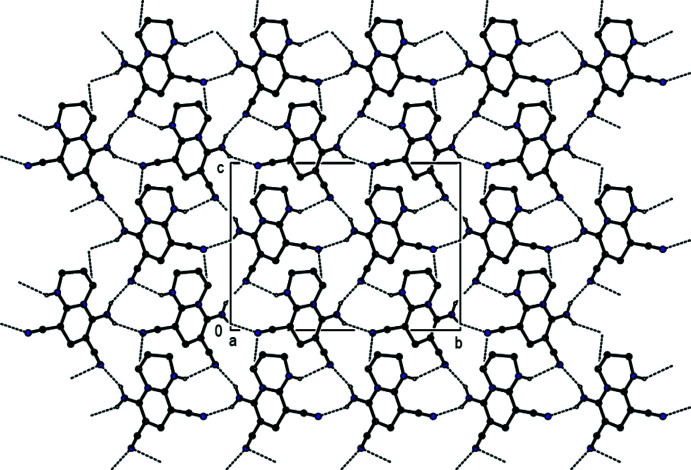
A view along [100] showing the inter­molecular N—H⋯N and C—H⋯N hydrogen bonds of the title compound. The di­chloro­phenyl group and H atoms not involved in hydrogen bonding have been omitted for clarity.

**Figure 4:**
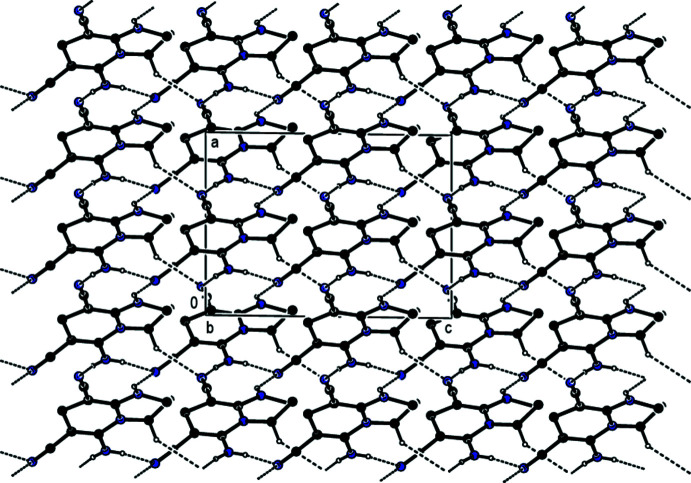
A view along [010] showing the inter­molecular N—H⋯N and C—H⋯N hydrogen bonds of the title compound. The di­chloro­phenyl group and H atoms not involved in hydrogen bonding have been omitted for clarity.

**Table 1:** Hydrogen-bond geometry (Å, °) *Cg*3 is the centroid of the C11–C16 di­chloro­phenyl ring.

*D*—H⋯*A*	*D*—H	H⋯*A*	*D*⋯*A*	*D*—H⋯*A*
N1—H1⋯N10^i^	0.85 (3)	2.43 (3)	3.152 (3)	143 (3)
N6—H6*A*⋯N17^ii^	0.85 (4)	2.17 (3)	2.927 (3)	149 (3)
N6—H6*B*⋯N10^iii^	0.85 (4)	2.16 (4)	2.953 (3)	156 (3)
C4—H4*B*⋯N17^iv^	0.99	2.59	3.440 (4)	144
C2—H2*A*⋯*Cg*3^iv^	0.99	2.87	3.653 (3)	136

Symmetry codes: (i) x+{\script{1\over 2}}, -y+{\script{3\over 2}}, z+{\script{1\over 2}}; (ii) x-{\script{1\over 2}}, y-{\script{1\over 2}}, z; (iii) x, -y+1, z+{\script{1\over 2}}; (iv) x-{\script{1\over 2}}, -y+{\script{3\over 2}}, z+{\script{1\over 2}}.

**Table 2:** Summary of short inter­atomic contacts (Å) in the title compound

Contact	Distance	Symmetry operation
H6*B*⋯N10	2.16	*x*, 1 − *y*, {1\over 2} + *z*
H1⋯N10	2.43	{1\over 2} + *x*, {3\over 2} − *y*, {1\over 2} + *z*
H4*B*⋯N17	2.59	−{1\over 2} + *x*, {3\over 2} − *y*, {1\over 2} + *z*
H6*A*⋯N17	2.16	−{1\over 2} + *x*, −{1\over 2} + *y*, *z*
N10⋯H15	2.81	−1 + *x*, *y*, *z*
H3*B*⋯H13	2.57	*x*, *y*, 1 + *z*

**Table 3:** Percentage contributions of inter­atomic contacts to the Hirshfeld surface for the title compound

Contact	Percentage contribution
N⋯H/H⋯N	28.4
H⋯H	24.5
C⋯H/H⋯C	21.4
Cl⋯H/H⋯Cl	16.1
Cl⋯C/C⋯Cl	3.3
Cl⋯Cl	2.5
Cl⋯N/N⋯Cl	2.3
C⋯N/N⋯C	0.8
C⋯C	0.6
N⋯N	0.2

**Table 4:** Experimental details

Crystal data	
Chemical formula	C_16_H_13_Cl_2_N_5_
*M*_r_	346.21
Crystal system, space group	Monoclinic, *C* *c*
Temperature (K)	100
*a*, *b*, *c* (Å)	8.6598 (2), 16.0275 (5), 11.6590 (3)
β (°)	90.7364 (9)
*V* (Å^3^)	1618.08 (8)
*Z*	4
Radiation type	Mo *K*α
μ (mm^−1^)	0.41
Crystal size (mm)	0.30 × 0.03 × 0.03

Data collection	
Diffractometer	Bruker D8 QUEST PHOTON-III CCD
Absorption correction	Multi-scan (*SADABS*; Krause *et al.,* 2015 ▸)
*T*_min_, *T*_max_	0.880, 0.980
No. of measured, independent and observed [*I* > 2σ(*I*)] reflections	21346, 5861, 4528
*R*_int_	0.064
(sin θ/λ)_max_ (Å^−1^)	0.758

Refinement	
*R*[*F* ^2^ > 2σ(*F* ^2^)], *wR*(*F* ^2^), *S*	0.044, 0.090, 1.03
No. of reflections	5861
No. of parameters	217
No. of restraints	2
H-atom treatment	H atoms treated by a mixture of independent and constrained refinement
Δρ_max_, Δρ_min_ (e Å^−3^)	0.25, −0.32
Absolute structure	Flack *x* determined using 1774 quotients [(*I* ^+^)−(*I* ^−^)]/[(*I* ^+^)+(*I* ^−^)] (Parsons *et al.*, 2013 ▸).
Absolute structure parameter	0.27 (3)

Computer programs: *APEX3* (Bruker, 2018 ▸), *SAINT* (Bruker, 2013 ▸), *SHELXT2014/5* (Sheldrick, 2015*a* ▸), *SHELXL2018/3* (Sheldrick, 2015*b* ▸), *ORTEP-3 for Windows* (Farrugia, 2012 ▸) and *PLATON* (Spek, 2020 ▸).

## supplementary crystallographic information

### Crystal data

**Table:**

C_16_H_13_Cl_2_N_5_	*F*(000) = 712
*M**_r_* = 346.21	*D*_x_ = 1.421 Mg m^−^^3^
Monoclinic, *C**c*	Mo *K*α radiation, λ = 0.71073 Å
*a* = 8.6598 (2) Å	Cell parameters from 4611 reflections
*b* = 16.0275 (5) Å	θ = 2.5–32.2°
*c* = 11.6590 (3) Å	µ = 0.41 mm^−^^1^
β = 90.7364 (9)°	*T* = 100 K
*V* = 1618.08 (8) Å^3^	Needle, colourless
*Z* = 4	0.30 × 0.03 × 0.03 mm

### Data collection

**Table:**

Bruker D8 QUEST PHOTON-III CCD diffractometer	4528 reflections with *I* > 2σ(*I*)
φ and ω scans	*R*_int_ = 0.064
Absorption correction: multi-scan (SADABS; Krause *et al.,* 2015)	θ_max_ = 32.6°, θ_min_ = 2.5°
*T*_min_ = 0.880, *T*_max_ = 0.980	*h* = −13→13
21346 measured reflections	*k* = −24→24
5861 independent reflections	*l* = −17→17

### Refinement

**Table:**

Refinement on *F*^2^	Secondary atom site location: difference Fourier map
Least-squares matrix: full	Hydrogen site location: mixed
*R*[*F*^2^ > 2σ(*F*^2^)] = 0.044	H atoms treated by a mixture of independent and constrained refinement
*wR*(*F*^2^) = 0.090	*w* = 1/[σ^2^(*F*_o_^2^) + (0.0315*P*)^2^ + 0.2854*P*] where *P* = (*F*_o_^2^ + 2*F*_c_^2^)/3
*S* = 1.02	(Δ/σ)_max_ < 0.001
5861 reflections	Δρ_max_ = 0.25 e Å^−^^3^
217 parameters	Δρ_min_ = −0.32 e Å^−^^3^
2 restraints	Absolute structure: Flack *x* determined using 1774 quotients [(*I*^+^)-(*I*^-^)]/[(*I*^+^)+(*I*^-^)] (Parsons et al., 2013).
Primary atom site location: difference Fourier map	Absolute structure parameter: 0.27 (3)

### Special details

**Table:**

Geometry. All esds (except the esd in the dihedral angle between two l.s. planes) are estimated using the full covariance matrix. The cell esds are taken into account individually in the estimation of esds in distances, angles and torsion angles; correlations between esds in cell parameters are only used when they are defined by crystal symmetry. An approximate (isotropic) treatment of cell esds is used for estimating esds involving l.s. planes.

### Fractional atomic coordinates and isotropic or equivalent isotropic displacement parameters (Å^2^)

**Table:**

	*x*	*y*	*z*	*U*_iso_*/*U*_eq_	
Cl1	0.48473 (9)	0.76604 (6)	0.16871 (7)	0.0429 (2)	
Cl2	0.69787 (9)	0.56058 (4)	0.51124 (7)	0.03080 (17)	
N1	0.5635 (3)	0.75406 (14)	0.7250 (2)	0.0199 (5)	
H1	0.604 (4)	0.801 (2)	0.709 (3)	0.024*	
C2	0.5218 (3)	0.73373 (16)	0.8418 (2)	0.0207 (5)	
H2A	0.4154	0.7528	0.8576	0.025*	
H2B	0.5934	0.7611	0.8969	0.025*	
C3	0.5326 (4)	0.63931 (17)	0.8531 (2)	0.0248 (6)	
H3A	0.6392	0.6206	0.8373	0.030*	
H3B	0.5064	0.6222	0.9321	0.030*	
C4	0.4209 (3)	0.59988 (16)	0.7681 (2)	0.0218 (5)	
H4A	0.4471	0.5401	0.7595	0.026*	
H4B	0.3151	0.6033	0.7990	0.026*	
N5	0.4232 (3)	0.64027 (13)	0.65394 (19)	0.0167 (4)	
C6	0.3418 (3)	0.60234 (14)	0.5649 (2)	0.0163 (5)	
N6	0.2511 (3)	0.53821 (15)	0.5930 (2)	0.0236 (5)	
H6A	0.214 (4)	0.506 (2)	0.541 (3)	0.028*	
H6B	0.238 (4)	0.522 (2)	0.661 (3)	0.028*	
C7	0.3535 (3)	0.63028 (15)	0.4540 (2)	0.0155 (5)	
C8	0.4667 (3)	0.69614 (15)	0.4164 (2)	0.0167 (5)	
H8	0.4066	0.7379	0.3704	0.020*	
C9	0.5266 (3)	0.74104 (16)	0.5222 (2)	0.0179 (5)	
C9A	0.5062 (3)	0.71289 (15)	0.6322 (2)	0.0161 (5)	
C10	0.2600 (3)	0.59393 (15)	0.3682 (2)	0.0147 (5)	
N10	0.1851 (3)	0.56730 (13)	0.2941 (2)	0.0194 (5)	
C11	0.5914 (3)	0.66149 (18)	0.3380 (3)	0.0200 (5)	
C12	0.6051 (3)	0.6880 (2)	0.2246 (3)	0.0283 (6)	
C13	0.7126 (4)	0.6549 (3)	0.1492 (3)	0.0375 (8)	
H13	0.7167	0.6742	0.0723	0.045*	
C14	0.8127 (4)	0.5940 (2)	0.1873 (3)	0.0383 (8)	
H14	0.8869	0.5715	0.1367	0.046*	
C15	0.8058 (3)	0.56565 (19)	0.2989 (3)	0.0314 (7)	
H15	0.8746	0.5235	0.3254	0.038*	
C16	0.6971 (3)	0.59938 (18)	0.3718 (3)	0.0240 (6)	
C17	0.5995 (3)	0.81835 (17)	0.5040 (2)	0.0228 (6)	
N17	0.6562 (4)	0.88151 (16)	0.4841 (2)	0.0362 (7)	

### Atomic displacement parameters (Å^2^)

**Table:**

	*U*^11^	*U*^22^	*U*^33^	*U*^12^	*U*^13^	*U*^23^
Cl1	0.0391 (4)	0.0681 (6)	0.0215 (4)	−0.0012 (4)	0.0009 (3)	0.0190 (4)
Cl2	0.0305 (4)	0.0267 (3)	0.0353 (4)	0.0081 (3)	0.0050 (3)	0.0077 (3)
N1	0.0294 (12)	0.0147 (10)	0.0156 (11)	−0.0072 (9)	0.0006 (9)	0.0002 (8)
C2	0.0278 (14)	0.0215 (13)	0.0127 (12)	−0.0055 (10)	−0.0012 (10)	−0.0013 (10)
C3	0.0353 (16)	0.0207 (13)	0.0180 (14)	−0.0050 (11)	−0.0071 (12)	0.0033 (10)
C4	0.0328 (14)	0.0189 (12)	0.0136 (13)	−0.0088 (11)	−0.0029 (11)	0.0027 (9)
N5	0.0237 (11)	0.0145 (10)	0.0120 (10)	−0.0050 (8)	0.0000 (8)	0.0003 (7)
C6	0.0200 (12)	0.0127 (10)	0.0161 (12)	−0.0016 (9)	−0.0006 (9)	−0.0018 (9)
N6	0.0354 (13)	0.0211 (11)	0.0142 (11)	−0.0141 (10)	−0.0024 (10)	0.0009 (9)
C7	0.0179 (11)	0.0150 (11)	0.0137 (12)	−0.0006 (9)	0.0007 (9)	−0.0013 (9)
C8	0.0216 (13)	0.0135 (10)	0.0151 (12)	−0.0022 (9)	0.0001 (10)	0.0002 (9)
C9	0.0242 (13)	0.0149 (11)	0.0148 (13)	−0.0040 (9)	0.0029 (10)	−0.0025 (9)
C9A	0.0189 (12)	0.0131 (10)	0.0163 (12)	−0.0025 (9)	0.0001 (9)	−0.0007 (9)
C10	0.0179 (11)	0.0123 (10)	0.0140 (12)	0.0010 (9)	0.0034 (9)	0.0018 (8)
N10	0.0226 (11)	0.0193 (11)	0.0163 (12)	0.0030 (9)	−0.0013 (9)	−0.0016 (8)
C11	0.0220 (12)	0.0199 (11)	0.0181 (12)	−0.0082 (9)	0.0022 (10)	−0.0051 (9)
C12	0.0257 (15)	0.0417 (17)	0.0175 (15)	−0.0130 (13)	0.0008 (11)	−0.0018 (12)
C13	0.0281 (16)	0.067 (2)	0.0179 (15)	−0.0197 (16)	0.0048 (12)	−0.0117 (15)
C14	0.0248 (15)	0.053 (2)	0.038 (2)	−0.0131 (15)	0.0123 (13)	−0.0255 (16)
C15	0.0214 (14)	0.0300 (15)	0.043 (2)	−0.0064 (12)	0.0079 (13)	−0.0150 (14)
C16	0.0228 (14)	0.0229 (13)	0.0264 (15)	−0.0039 (10)	0.0037 (11)	−0.0039 (11)
C17	0.0329 (15)	0.0213 (12)	0.0144 (13)	−0.0073 (11)	0.0054 (11)	−0.0053 (10)
N17	0.064 (2)	0.0259 (12)	0.0189 (13)	−0.0208 (13)	0.0093 (12)	−0.0046 (10)

### Geometric parameters (Å, º)

**Table:**

Cl1—C12	1.749 (4)	N6—H6B	0.85 (4)
Cl2—C16	1.741 (3)	C7—C10	1.406 (4)
N1—C9A	1.355 (3)	C7—C8	1.509 (3)
N1—C2	1.451 (3)	C8—C9	1.514 (4)
N1—H1	0.85 (3)	C8—C11	1.529 (4)
C2—C3	1.522 (4)	C8—H8	1.0000
C2—H2A	0.9900	C9—C9A	1.373 (4)
C2—H2B	0.9900	C9—C17	1.408 (4)
C3—C4	1.514 (4)	C10—N10	1.155 (3)
C3—H3A	0.9900	C11—C12	1.395 (4)
C3—H3B	0.9900	C11—C16	1.405 (4)
C4—N5	1.481 (3)	C12—C13	1.392 (4)
C4—H4A	0.9900	C13—C14	1.375 (5)
C4—H4B	0.9900	C13—H13	0.9500
N5—C6	1.387 (3)	C14—C15	1.380 (5)
N5—C9A	1.393 (3)	C14—H14	0.9500
C6—N6	1.337 (3)	C15—C16	1.385 (4)
C6—C7	1.373 (4)	C15—H15	0.9500
N6—H6A	0.85 (4)	C17—N17	1.150 (3)
			
C9A—N1—C2	123.1 (2)	C7—C8—C9	108.2 (2)
C9A—N1—H1	114 (2)	C7—C8—C11	112.7 (2)
C2—N1—H1	121 (2)	C9—C8—C11	115.0 (2)
N1—C2—C3	106.8 (2)	C7—C8—H8	106.8
N1—C2—H2A	110.4	C9—C8—H8	106.8
C3—C2—H2A	110.4	C11—C8—H8	106.8
N1—C2—H2B	110.4	C9A—C9—C17	119.5 (2)
C3—C2—H2B	110.4	C9A—C9—C8	123.9 (2)
H2A—C2—H2B	108.6	C17—C9—C8	116.4 (2)
C4—C3—C2	108.7 (2)	N1—C9A—C9	122.4 (2)
C4—C3—H3A	110.0	N1—C9A—N5	116.5 (2)
C2—C3—H3A	110.0	C9—C9A—N5	121.1 (2)
C4—C3—H3B	110.0	N10—C10—C7	176.6 (3)
C2—C3—H3B	110.0	C12—C11—C16	114.7 (3)
H3A—C3—H3B	108.3	C12—C11—C8	121.7 (3)
N5—C4—C3	113.0 (2)	C16—C11—C8	123.5 (3)
N5—C4—H4A	109.0	C13—C12—C11	123.2 (3)
C3—C4—H4A	109.0	C13—C12—Cl1	115.9 (3)
N5—C4—H4B	109.0	C11—C12—Cl1	120.8 (2)
C3—C4—H4B	109.0	C14—C13—C12	119.3 (3)
H4A—C4—H4B	107.8	C14—C13—H13	120.3
C6—N5—C9A	119.3 (2)	C12—C13—H13	120.3
C6—N5—C4	117.9 (2)	C13—C14—C15	120.2 (3)
C9A—N5—C4	122.8 (2)	C13—C14—H14	119.9
N6—C6—C7	122.1 (2)	C15—C14—H14	119.9
N6—C6—N5	116.6 (2)	C14—C15—C16	119.2 (3)
C7—C6—N5	121.2 (2)	C14—C15—H15	120.4
C6—N6—H6A	120 (2)	C16—C15—H15	120.4
C6—N6—H6B	124 (2)	C15—C16—C11	123.3 (3)
H6A—N6—H6B	115 (3)	C15—C16—Cl2	116.0 (3)
C6—C7—C10	119.1 (2)	C11—C16—Cl2	120.6 (2)
C6—C7—C8	123.9 (2)	N17—C17—C9	176.9 (3)
C10—C7—C8	117.0 (2)		
			
C9A—N1—C2—C3	46.5 (3)	C17—C9—C9A—N5	174.5 (3)
N1—C2—C3—C4	−60.3 (3)	C8—C9—C9A—N5	−1.8 (4)
C2—C3—C4—N5	43.2 (3)	C6—N5—C9A—N1	170.2 (2)
C3—C4—N5—C6	171.2 (2)	C4—N5—C9A—N1	−11.5 (4)
C3—C4—N5—C9A	−7.1 (4)	C6—N5—C9A—C9	−9.1 (4)
C9A—N5—C6—N6	−173.0 (2)	C4—N5—C9A—C9	169.2 (2)
C4—N5—C6—N6	8.6 (4)	C7—C8—C11—C12	116.4 (3)
C9A—N5—C6—C7	6.4 (4)	C9—C8—C11—C12	−118.9 (3)
C4—N5—C6—C7	−172.0 (2)	C7—C8—C11—C16	−61.5 (3)
N6—C6—C7—C10	4.1 (4)	C9—C8—C11—C16	63.2 (3)
N5—C6—C7—C10	−175.2 (2)	C16—C11—C12—C13	1.1 (4)
N6—C6—C7—C8	−173.2 (2)	C8—C11—C12—C13	−176.9 (3)
N5—C6—C7—C8	7.4 (4)	C16—C11—C12—Cl1	−179.0 (2)
C6—C7—C8—C9	−16.0 (3)	C8—C11—C12—Cl1	3.0 (4)
C10—C7—C8—C9	166.6 (2)	C11—C12—C13—C14	−1.0 (5)
C6—C7—C8—C11	112.3 (3)	Cl1—C12—C13—C14	179.1 (2)
C10—C7—C8—C11	−65.1 (3)	C12—C13—C14—C15	0.5 (5)
C7—C8—C9—C9A	13.2 (3)	C13—C14—C15—C16	−0.2 (5)
C11—C8—C9—C9A	−113.8 (3)	C14—C15—C16—C11	0.4 (4)
C7—C8—C9—C17	−163.2 (2)	C14—C15—C16—Cl2	−179.4 (2)
C11—C8—C9—C17	69.8 (3)	C12—C11—C16—C15	−0.8 (4)
C2—N1—C9A—C9	169.2 (3)	C8—C11—C16—C15	177.2 (3)
C2—N1—C9A—N5	−10.1 (4)	C12—C11—C16—Cl2	179.0 (2)
C17—C9—C9A—N1	−4.7 (4)	C8—C11—C16—Cl2	−3.1 (4)
C8—C9—C9A—N1	179.0 (3)		

### Hydrogen-bond geometry (Å, º)

*Cg*3 is the centroid of the C11–C16 dichlorophenyl ring.

**Table:**

*D*—H···*A*	*D*—H	H···*A*	*D*···*A*	*D*—H···*A*
N1—H1···N10^i^	0.85 (3)	2.43 (3)	3.152 (3)	143 (3)
N6—H6*A*···N17^ii^	0.85 (4)	2.17 (3)	2.927 (3)	149 (3)
N6—H6*B*···N10^iii^	0.85 (4)	2.16 (4)	2.953 (3)	156 (3)
C4—H4*B*···N17^iv^	0.99	2.59	3.440 (4)	144
C2—H2*A*···*Cg*3^iv^	0.99	2.87	3.653 (3)	136

Symmetry codes: (i) *x*+1/2, −*y*+3/2, *z*+1/2; (ii) *x*−1/2, *y*−1/2, *z*; (iii) *x*, −*y*+1, *z*+1/2; (iv) *x*−1/2, −*y*+3/2, *z*+1/2.
